# Role of Oritavancin in the Treatment of Infective Endocarditis, Catheter- or Device-Related Infections, Bloodstream Infections, and Bone and Prosthetic Joint Infections in Humans: Narrative Review and Possible Developments

**DOI:** 10.3390/life13040959

**Published:** 2023-04-06

**Authors:** Tommaso Lupia, Ilaria De Benedetto, Roberta Bosio, Nour Shbaklo, Francesco Giuseppe De Rosa, Silvia Corcione

**Affiliations:** 1 Unit of Infectious Diseases, Cardinal Massaia, 14100 Asti, Italy; 2 Department of Medical Sciences, Infectious Diseases, University of Turin, 10126 Turin, Italy; 3 School of Medicine, Tufts University, Boston, MA 02111, USA

**Keywords:** oritavancin, long-acting, Gram-positive, vascular infections, prosthetic infections, device infections, enterococcal infections, review

## Abstract

Oritavancin is a long-acting lipoglycopeptide with in vitro activity against Gram-positive pathogens, as well as good bactericidal activity and sterilisation ability in biofilm. It has been approved for acute bacterial skin and skin structure infections (ABSSSI), but recent reports have demonstrated possible off-label uses, such as for vancomycin resistant enterococci (VRE), deep-seated infections including those involving prosthetic material and invasive infections. The aim of this work is to review the uses of oritavancin outside of ABSSSI, focusing on its real-life applications on infective endocarditis, catheter- or device-related infections, bloodstream infections, and bone and prosthetic joint infections in humans, as well as possible future applications. We performed a narrative review, collecting the literature published between 1 December 2002 and 1 November 2022 on PubMed and the Cochrane Library using the term ‘oritavancin’. Available studies have shown how effective it is in different settings, suggesting an opportunity for step-down strategies or outpatient management of infections requiring a long duration of antibiotic treatment. So far, evidence is still scarce, and limited to a few studies and case reports, mostly focusing on *Staphylococcus aureus* as the major isolate. Concerns about fluid intake for dilution and interaction with coagulation markers also need to be taken into account. Further studies are required in order to assess the safety and effectiveness of Oritavancin in vascular, prosthetic, or device-related infections, as well as in resistant Gram-positive bacteria or enterococcal infections.

## 1. Introduction

Oritavancin diphosphate (oritavancin) is a semi-synthetic, long-acting lipoglycopeptide (LGP) with potent activity against Gram-positive pathogens, such as methicillin-resistant *Staphylococcus aureus* (MRSA); vancomycin-intermediate *S. aureus* (VISA); hetero-resistant VISA (hVISA); vancomycin-resistant *S. aureus* (VRSA); Daptomycin-resistant *S. aureus*; vancomycin-resistant enterococci (VRE), including both Van A and Van B productor strains; streptococci (including *S. dysgalactiae*, *S. anginosus*, *S. intermedius* and *S. constellatus*); and several Gram-positive anaerobic bacteria [1,2].

Oritavancin has been approved in recent years by the Food and Drug Administration (FDA) and the European Medicines Agency (EMA) for acute bacterial skin and skin structure infections (ABSSSI) [3,4].

This molecule has three mechanisms of action: (i) the inhibition of cell wall biosynthesis (tranglycosylation) by binding to the peptide stem of peptidoglycan precursors; (ii) the inhibition of the transpeptidation (cross-linking) stage of cell wall biosynthesis by binding to the peptide bridges of the cell wall; (iii) and the disruption of the integrity of the bacterial membrane, resulting in depolarisation, permeabilisation and rapid cell death [1,2].

Oritavancin is administered as a single 1200 mg intravenous (IV) infusion over 3 h [3,4]. The package comprises three single-use vials, each containing lyophilised oritavancin (400 mg) and an inactive component, mannitol. Subsequently, the vials are reconstituted with sterile water for infusion (SWFI) and further diluted in 5% dextrose, 5% sterile water (D5W) for a total volume of 1000 mL [3,4]. Recently, Hoover et al. [5] described a new formulation of oritavancin (Kimyrsa^®^) that can be infused in 250 mL of D5W or normal saline solution (NS). It was also developed to shorten the time of infusion from 3 h to 1 h. Therefore, this new formulation, currently not available in Europe and available mainly in United States, simplifies the preparation of the solution and increases flexibility, especially in patients with congestive heart failure or insulin-dependent diabetes mellitus. Despite that, Kimyrsa^®^ includes within the excipients 2-hydroxypropyl-Beta-cyclodestrin, which can increase the risk of nephrotoxicity [5]. Moreover, no dosage adjustment of Kimyrsa^®^ is needed in patients with mild or moderate renal impairment and the pharmacokinetics in severe renal impairment have not been evaluated [5].

The single-dose infusion is made possible by its mainly concentration-dependent activity and prolonged half-life, and this provides an alternative to multi-dose daily therapies, allowing earlier discharges [6]. Since lipoglycopeptide and lipopeptide antimicrobial molecules interfere with some phospholipid-dependent coagulation markers, oritavancin has been shown to alter some coagulation tests, artificially modifying prothrombin time (PT), partial thromboplastin activated time (aPTT), and other tests for more than 120 h after infusion [7]. The interference of oritavancin in these tests is temporary, and the results revert to normal ranges within a few days after dosing [1,7].

In the registrative non-inferiority double-blind trials in Gram-positive pathogen-sustained ABSSSI (SOLO I and SOLO II) [8,9], patients were randomised to receive either a single intravenous dose of 1200 mg of oritavancin or intravenous vancomycin twice daily for 7 to 10 days. The primary endpoint was considered the cessation of the spreading of, or reduction in, lesion size; the absence of fever; and no need for administration of a rescue antibiotic 48 to 72 h after oritavancin. Secondary endpoints were a clinical cure 7 to 14 days after the end of treatment and a reduction in lesion size of 20% or more 48 to 72 h after the administration of oritavancin. All endpoints were met for all considered pathogens, including methicillin-resistant *Staphylococcus aureus* [8,9].

Recent studies have demonstrated possible off-label uses, such as for VRE, deep-seated or invasive infections, including in patients with bloodstream infections (BSIs), and bone and joint infections (BJIs), and other authors have attempted to summarize some evidence regarding possible off-label uses of oritavancin [10]. The aim of this work is to review the uses of oritavancin outside of ABSSSI, focusing on infective endocarditis, catheter- or device-related infections, bloodstream infections, and bone and prosthetic joint infections, as well as the possible future applications of this molecule. This is the first narrative review, to our knowledge, focusing on oritavancin only, and its potential off-label uses and possible future developments.

## 2. Materials and Methods

The current narrative review followed five steps: identifying the research question, search methods to identify relevant studies, study selection, charting and summarising data, and reporting the results. Moreover, we have followed the recommendations provided by the Scale for the Assessment of Narrative Review Articles (SANRA) for reporting narrative reviews [11].

The main research question was to summarise current evidence on oritavancin in infective endocarditis, catheter- or device-related infections, bloodstream infections, and prosthetic joint infections in humans. A search was run on PubMed and the Cochrane Library using the term ‘oritavancin’ in English. The results were limited to between 1 December 2002 and 1 November 2022.

**Figure 1:**
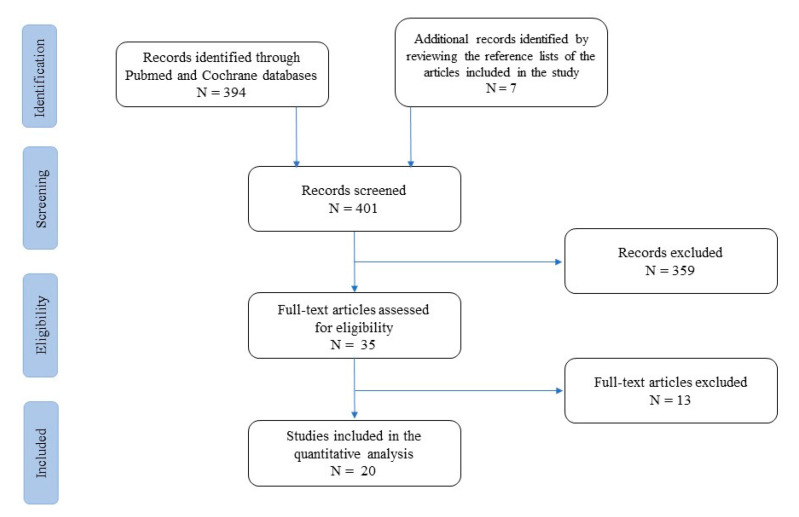
Flow-chart of studies included and excluded from the narrative review.

At first, studies were grouped by practice guidelines, guidelines, meta-analyses, systematic reviews, narrative reviews, case series, and case reports (Figure 1).

A list of 394 papers was generated from the initial search. The reviewers then studied the titles and abstracts. After this review, thirty-five papers were included.

Two independent reviewers (TL and IDB) reviewed the titles and summaries of all articles sought and used data from 22 full articles to compile this review paper. We included papers that described evidence on oritavancin use in infective endocarditis, catheter- or device-related infections, bloodstream infections, and prosthetic joint infections. We excluded papers with no clear methods, duplicated works of previously included papers, and papers which did not utilize the English language. The results were reported in five categories and eventually analysed to provide a critical discussion and to identify knowledge, gaps, and novel insights.

## 3. Results

### 3.1. Oritavancin in Infective Endocarditis

Real-life examples of treating infective endocarditis (IE) with oritavancin emerged from the literature. The first report, by Johnson et al. [12], described a case of recurrent vancomycin-resistant enterococcus (VRE) bacteraemia due to mitral prosthetic valve endocarditis that was treated with an attack dose of 1200 mg every 48 h for three consecutive doses, followed by 1200 mg twice weekly for six weeks. In this patient, oritavancin was initiated after microbiological failure with a combination therapy of daptomycin plus tigecycline, followed by linezolid plus tigecycline. Eight days afterward, the oritavancin regimen was stopped, and the blood culture turned positive for VRE with similar susceptibility test results as presented previously. Subsequently, oritavancin was restarted at 1200 mg biweekly for 10 weeks. This patient needed valve replacement surgery in combination with antibiotic surgery, which determined a favourable outcome. In addition, before surgery, tigecycline and linezolid were restarted until 10 days post-surgical intervention [12]. Moreover, Stewart et al. reported a comprehensive real-life application of oritavancin [13]. This work illustrated a case of native tricuspid IE and shoulder myositis due to *Streptococcus agalactiae* treated with a single dose (1200 mg) of step-down therapy with oritavancin after a short treatment with ceftriaxone and vancomycin, which resulted in a clinical failure, defined as the need for surgical valve replacement three months later [13]. More recently, Brownell et al. described a more comprehensive case series of infective endocarditis treated with oritavancin that resulted in clinical success, despite the authors not reporting microbiological aetiologies of IE or adverse events in these patients following oritavancin infusion [14]. Other endocarditis case reports and case series worth mentioning are summarised in Table 1 [12,13,14,15,16,17].

Notably, a recent registered clinical trial (NCT03761953) that comprised IE cases was withdrawn. It was designed as a single-centre, open-label pilot study regarding the use of oritavancin in *S. aureus* bacteraemia (with or without IE) that focused on opioid users [26]. Unfortunately, this study, endorsed by the University of Pennsylvania, ceased due to the COVID-19 pandemic.

### 3.2. Oritavancin in Catheter- or Device-Related Infections

Oritavancin is a promising treatment option in patients with Gram-positive infections related to intravascular devices, such as short- or long-term vascular catheters and vascular prostheses, due to the long half-life of this molecule and weekly dosing that provides clear, practical advantages in treating infections that need long-term antibiotic therapies [1,10]. Unfortunately, the literature in this field is still scarce, and few case reports are available. Stewart et al. described a catheter-related MSSA bacteraemia that was successfully treated with a single dose of oritavancin after a short course of cefazolin (one day) and vancomycin (four days) with a complete cure and removal of the PICC line [13]. In a multicentre retrospective analysis, Morrisette et al. collected two cases of catheter-related BSI treated with oritavancin [16]. Interestingly, Schulz et al. reported a single case of endovascular graft infection due to *Staphylococcus lugdunensis* treated with a multidose scheme (sequentially: 1200 mg; 800 mg/wk × 11 doses; 1200 mg following 11-day intervals; and 800 mg × 5 doses/week) with clinical improvement [18]. Oritavancin was chosen as a palliative, suppressive treatment following successful treatment with cefazolin. However, the graft could not be removed, and the patient was not a surgical candidate [18].

Prolonged dosing regimens have resulted in cure for patients with first-line treatment failure. While robust evidence is needed in order to demonstrate the efficacy and safety of continued dosing of oritavancin, available studies may fill an important treatment niche in this era of growing resistance [27].

So far, the efficacy of oritavancin in continued dosing was not assessed in randomised controlled trials, but several studies show promising outcomes [12,13,14,15,16,17,18,26]. Data from the Clinical and Historic Registry and Orbactiv Medical Evaluation (CHROME) patient registry included 32 patients receiving multiple oritavancin doses for Gram-positive infections with an overall 93.8% success rate [20].

Warren Rose et al. studied the pharmacokinetic estimates for a 1200 mg single dose with and without an 800 mg dose 1 week apart [28]. The dosing of oritavancin showed predictable linear pharmacokinetics and therapeutic concentrations. Oritavancin concentrations (total and free) stayed above the sensitivity breakpoint (0.12 mg/L) for 8 weeks and 4.6 weeks, respectively, with the two-dose regimen [28]. This regimen resulted in a greater area under the drug concentration–time curve (AUC) and above the sensitivity breakpoint in comparison to the single-dose regimen.

Interestingly, Carvalhaes et al. [29] evaluated the in vitro activity of oritavancin and comparators against coagulase-negative staphylococci (CoNS) in BSIs. In total, 587 CoNS isolates (1/patient) were collected. Identification was performed by MALDI-TOF, and susceptibility testing was performed using CLSI broth microdilution methodology. Oritavancin was greatly active and inhibited more than 96% of all CoNS and individual species (>10 isolates) at ≤0.12 mg/L, independently of a methicillin profile, except for *S. haemolyticus*. These data should be of interest in catheter-related infections due to the high incidence of CoNS infections [29].

Regarding cardiac device infections, it is worth mentioning the applications of oritavancin reported by Co et al. in a collection of seven device infections [19]. Other significant device or vascular graft case reports are summarised in Table 1.

### 3.3. Oritavancin in Isolated Blood-Stream Infections

Oritavancin use was evaluated in Gram-positive bacteraemia during the first years of its development. In 2006, Bhavnani et al. [30] reported data from patients with uncomplicated *S. aureus* bacteraemia who were randomly assigned to receive either oritavancin or standard-of-care therapy with beta-lactam or vancomycin (for MSSA or MRSA, respectively). Patients in this phase 2 randomised study were assigned to receive oritavancin at a dose ranging from 5 to 10 mg/kg daily [30]. This contrasts with the fixed-dose, prolonged-interval techniques now being approved. Oritavancin was given to 86 individuals, although only 55 could be evaluated for microbiological and clinical responses [17]. Both clinical and microbiological success were reported, in 47 (85%) and 45 (78%) patients, respectively [30]. Exploratory pharmacokinetic and pharmacodynamic analyses revealed a tenuous relationship between clinical success and the percentage of time the free drug was above the MIC [30]. Moreover, experience with oritavancin as a treatment alternative for bacteraemia is restricted to case reports and short series, except for this clinical investigation [13,17,19]. The available data originate from patients infected with a wide variety of Gram-positive pathogens, the vast majority of which involve staphylococci, enterococci, and streptococci. Significantly, oritavancin has been utilised almost exclusively as a consolidation regimen in patients previously handled with other antimicrobials. Other case reports and case series involving oritavancin use in bloodstream infections are summarised in Table 1.

### 3.4. Oritavancin in Bone and Prosthetic Joint Infections

Oritavancin has shown favourable PK/PD, wide distribution volume, good bone penetration, in vitro bactericidal activity against stationary-phase *S. aureus* cells, and the sterilisation of biofilms [31]. Correspondingly, it could represent a particularly appealing molecule for treating osteomyelitis, including cases involving prosthetic devices. In an in vitro study, oritavancin was tested against 185 staphylococci isolates associated with prosthetic joint infection, of which 37 were MRSA, 67 were MSSA, 59 were methicillin-resistant *S. epidermidis* (MRSE), and 22 were methicillin-susceptible *S. epidermidis* (MSSE). The oritavancin MIC_50_ for *S. aureus* and MSSE was 0.03 μg/mL, and for MRSE, it was 0.06 μg/mL. MIC_90_ for *S. aureus* and *S. epidermidis* was 0.12 μg/mL for both the methicillin-resistant and -susceptible subgroups. The oritavancin MBBC_50_ for *S. aureus* and *S. epidermidis* was 2 μg/mL for both the methicillin-resistant and -susceptible subgroups. The MBBC_90_ for *S. aureus* and MSSE was 4 μg/mL, and for MRSE, it was 8 μg/mL [32].

There are studies [18,19,21] and case reports [22,23,24,25] describing off-label use in bone and prosthetic-associated infections. A multicentre retrospective study described 134 patients with acute osteomyelitis who obtained clinical success in 88.1% of cases—defined as the resolution of symptoms or improvement of symptoms and no further need for treatment—after receiving four or five doses of oritavancin (1200 mg, then 800 mg weekly). In most cases, MRSA was the causative pathogen (71.9%), and a small percentage (6.7%) had a concomitant MRSA bloodstream infection. Overall, 17.9% were associated with prosthetic material, and surgical debridement was performed in 90.3% of cases [21]. Another observational cohort of 438 patients who received at least one dose of oritavancin included 18 cases of osteomyelitis, of which three involved prosthetic material. Cures or improvements after 30 days were achieved in 93.8% of cases [19]. Among case reports, Chastain et al. presented nine patients who received at least two doses of oritavancin in a multidose strategy for the treatment of chronic osteomyelitis and demonstrated a clinical cure at the 6-month follow-up after the last dose of oritavancin [23]. Oritavancin also displays activity against *Enterococcus* spp. In addition, case reports of its sequential use in bone and prosthetic-associated infections have started to emerge as a simplification strategy after initial treatment with daptomycin plus ampicillin [24], or combined with ampicillin in the case of vanA-producing vancomycin-resistant *Enterococcus faecium* (VRE) device-associated vertebral osteomyelitis [24,25], even though the drug is approved only for vancomycin-susceptible *Enterococcus faecalis*. To date, no clinical trials have been registered on clinicaltrial.gov for oritavancin use in bone or prosthetic-associated joint infections.

### 3.5. Oritavancin and Biofilm

Some in vitro studies have illustrated that oritavancin in combination with rifampin, gentamicin, moxifloxacin, linezolid, and β-lactams reveals synergistic activity against planktonic *S. aureus* [31,33,34]. The antibiofilm activity of oritavancin has been evaluated in combination with rifampin, gentamicin, or linezolid against 10 prosthetic joint infections sustained by MRSA isolates using time-kill assays. Its combination with rifampin demonstrated significant bacterial reductions compared with all other antimicrobials alone for 100% of the isolates, and synergy was observed in 80% of the isolates [34]. Similarly, Yan et al. tested the in vitro activity of oritavancin in combination with rifampin or gentamicin against 20 MRSE isolates from prosthetic joint infection biofilms. At 24 h, the combination of oritavancin and rifampin resulted in a significant reduction of biofilm density compared with all other antimicrobials alone for 85% of isolates vs. 55% for the combination of oritavancin and gentamicin. Synergy was observed against 65% of the isolates with oritavancin plus rifampin and 35% with oritavancin plus gentamicin [35]. Regarding *Enterococcus* spp., the in vitro activity of oritavancin against 60 vancomycin-susceptible enterococci and 27 vancomycin-resistant enterococci, both in planktonic and biofilm states, was evaluated. Oritavancin MIC ranged from ≤0.002 to 0.5 μg/mL with the minimum biofilm bactericidal concentration ranging from ≤0.002 to 2 μg/mL [36]. Lagatolla et al. tested five vanA and five vanB isolates of *Enterococcus faecium* using a combination of oritavancin and fosfomycin, and observed a synergistic effect in 80% of isolates and a restoration of fosfomycin susceptibility in 85% of fosfomycin-resistant isolates [37].

Moreover, antagonism was not observed between oritavancin and gentamicin, moxifloxacin, linezolid, or rifampin in in vitro studies [1].

## 4. Discussion

In this review, we summarised the current evidence reported in the literature regarding potential off-label uses of oritavancin in IE, device-related infections (including CLABSI, vascular grafts, and cardiac devices), BSI, and prosthetic-associated infections. In addition, we summarised the data on the biofilm activity of this antimicrobial molecule. Oritavancin appears to be a new promising molecule in the armamentarium of antibiotics for the treatment of Gram-positive sustained infections, with some possible extended applications. A single infusion may facilitate home discharge, intravenous access and removal, and compliance to therapy, reinforcing the opportunity of lineless antibiotics [1,38]. The term “lineless antibiotics” was coined in reference to long-life, intravenous antimicrobial molecules that do not need the long-term maintenance of intravenous access. IE, device-related infections, and isolated BSI are theoretical fields of interest for which new applications of oritavancin may develop in the near future.

**Figure 2:**
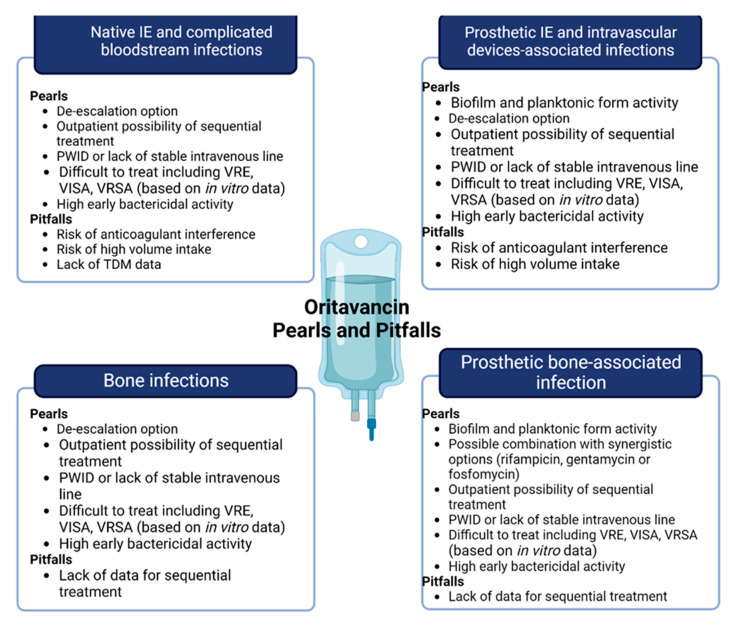
Pearls and pitfalls of oritavancin in off-label indication, including central boxes of “microbiology” or “step-down” and “sequential”. Abbreviations: VRE: vancomycin-resistant *Enterococcus*; VISA: Vancomycin-intermediate *Staphylococcus aureus*; VRSA: Vancomycin-resistant *Staphylococcus aureus*; PWID: people who inject drugs; TDM: therapeutic drug monitoring.

There are heterogeneous data on IE, with few patients collected, and mainly from case series and case reports (Table 1). A promising single-centre, open-label pilot study regarding the use of oritavancin against *S. aureus* bacteraemia (with or without IE) was unfortunately stopped during the first pandemic wave; no news on this study is available [26]. Existing examples are mainly represented by step-down therapies after initial antibiotic treatments with other anti-Gram-positive drugs (i.e., vancomycin, daptomycin, linezolid). Due to these biases, we cannot outline the correct clinical or microbiological window for oritavancin therapy in IE patients, beyond the clinical opportunities of long-acting therapy. We speculate that, in patients without local or systemic IE complications or surgical indications, oritavancin may represent an early step-down therapy wherein susceptible micro-organisms are isolated (Figure 2).

The microbiological point of view is interesting, as illustrated in Table 1. Oritavancin application was experimentally utilized against different microbiological isolates, including staphylococci, enterococci, and streptococci, with mostly favourable outcomes. Most of the clinical failures were associated with a lack of effective (surgical) source control, especially in PVE and IE with systemic complications (e.g., abscesses). Interestingly, Pfaller et al. [39] evaluated the microbiological profile of oritavancin in vitro within a wide group of bloodstream isolates recovered from IE patients in 2008 in the United States and Europe. Oritavancin showed potent activity against *S. aureus* and CoNS (98.8% in both of the groups when the isolates presented MIC ≤ 0.12 µg/mL of oritavancin) and enterococci (98.1%, including vancomycin-resistant isolates at ≤0.12 µg/mL of oritavancin) and reached 100% susceptibility in the viridans Streptococci group [39]. In addition, the authors concluded that oritavancin coverage against this Gram-positive collection was comparable to that of other agents, including daptomycin, linezolid, teicoplanin, and vancomycin. Moreover, in the studies summarised in Table 1, adverse events after infusion, including repeated treatment, were very rarely reported (e.g., hypoglycaemia, tachycardia, and eosinophilia).

However, some concerns should be considered for the cardiovascular population of patients with prosthetic valves, patients in need of classical oral direct anticoagulants (i.e., warfarin or acenocumarol), and patients with known coagulopathy with the need for strictly laboratory follow-up, due to potential drug interactions with oritavancin-containing regimens; despite that changes in coagulation laboratory tests were present in in vitro studies, and at this moment, given the design of most included studies, current data do not permit us to postulate a conclusion regarding the clinical relevance of the risk of bleeding. Moreover, the fluid intake requested for oritavancin intravenous administration may be a relative contraindication for patients at high risk of fluid overloading. The new formulation Kymirsa^®^ may be a possible solution to this problem, with a fluid intake of only 250 mL versus 1000 mL. Nonetheless, vials of Kymirsa^®^ may be less comfortable for sequential treatments due to the use of a single dosage (1200 mg) instead of the fractioned vials (400 mg) of the classical oritavancin treatment.

According to the lineless property of oritavancin, patients who need antibiotic treatment without a stable IV line, as, for example, patients with a known IV line infection, or patients with a scarce peripheral venous heritage (i.e., people who inject drugs—PWID—or obese people) should be considered for oritavancin treatment. In fact, many reports have included PWID [17,20] or patients with IV line infections (Table 1). Source control of the suspected infected IV line, or vascular device or hardware, is of central importance for oritavancin use, because failures are reported in the literature for patients without surgical debridement or in which the source of infection may not be resolved (Table 1).

Oritavancin has shown adequate in vitro bactericidal activity and sterilisation ability of biofilms, and some retrospective applications have described successful use in bone and prosthetic-associated infections as well [19,21,24,25], including one case with retained prosthetic materials [24]. In vitro studies have shown the ability of oritavancin to accumulate extensively in macrophages, potentially enhancing the eradication of pathogens that survive in lysosomes [1]. Some remaining questions need to be pointed out. First, the vast majority of cases in the existing literature on this kind of infection regard staphylococcal aetiology with a prevalence of methicillin-resistant strains. In this particular context, oritavancin has demonstrated effectiveness in sequential weekly administration schemes. Despite an adequate proven efficacy with a high rate of clinical success in some reports (Table 1) and in vitro bactericidal and synergic activity with rifampin or gentamicin [34,35], to date, no definitive data about the preferable dose or the number of doses are available for sequential use in the treatment of osteomyelitis and prosthetic joint infections. However, taken together, these studies seem to reveal that the more doses are administered, the more side effects are reported—even if mild, as mentioned previously in the case of IE—some of which have led to the discontinuation of the drug.

An unexplored, potentially interesting, field is the use of oritavancin in Gram-positive sustained infections with limited therapeutic options, such as vancomycin-intermediate *S. aureus* (VISA), heteroresistant VISA (hVISA), vancomycin-resistant *S. aureus* (VRSA), *Enterococcus* spp., and especially in vancomycin-resistant *Enterococcus faecium*. Oritavancin’s long-acting profile, together with its activity against enterococcal infections, could warrant a major role as a step-down option for outpatient treatment of infections that normally require several weeks of antibiotic therapy, such as IE and bone and prosthetic-associated infections, with or without retention. Nonetheless, in the registrative SOLO I trial [8], no cases of vancomycin, VISA, hVISA, or VRSA were included, while only 12 total cases of *Enterococcus faecalis*-sustained ABSSSI were included: 2 wound infections, 4 cellulites/erysipelas cases, and 6 major cutaneous abscesses. Moreover, in the small subgroup of *Enterococcus faecalis* wound infections, no patients were present in the vancomycin control group or in part of the two available case reports. Further in vivo studies are needed in order to assess the effectiveness of oritavancin against pathogens other than MSSA and MRSA, including not only *Enterococcus faecalis,* but also van-A-producing vancomycin-resistant *Enterococcus faecium*. In addition, since in vitro rapid early bactericidal activity has been demonstrated, this finding needs to be confirmed by in vivo studies, especially when used in high-inoculum infections or critical patients with bloodstream infections

**Figure 3:**
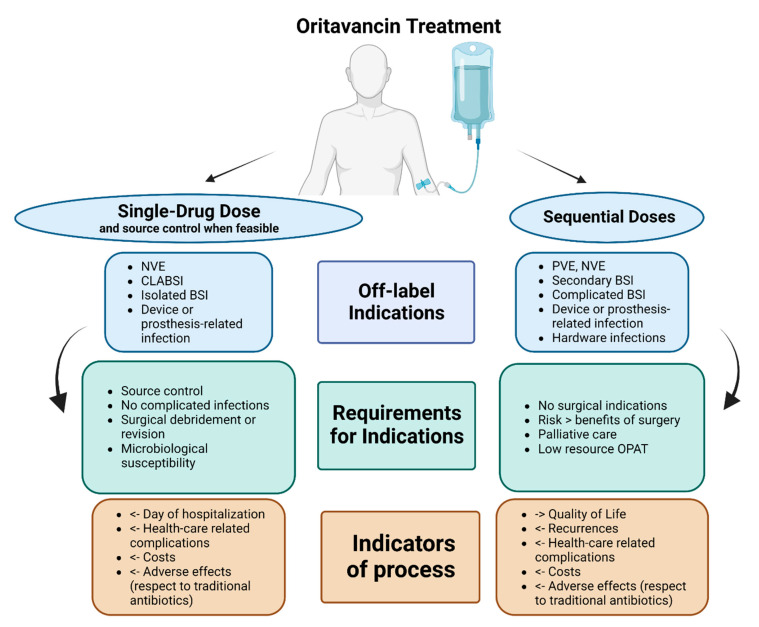
Authors’ proposal for place in therapy of oritavancin as single-drug dose or sequential doses in potential off-label indications (limited evidence available). Abbreviations: NVE: native valve endocarditis; PVE: prosthetic valve endocarditis; CLABSI: central-line-associated bloodstream infections; BSI: bloodstream infections; OPAT: Outpatient antibiotic treatment.

We summarised, in Figure 3, our proposal for the possible uses of oritavancin with curative or suppressive purpose, according to off-label indications, including requirements such as source control or surgical debridement, as well as stewardship indicators.

## 5. Conclusions

Available studies have shown the effective use of oritavancin in different settings, suggesting an opportunity for step-down or sequential treatment strategies, including the management of outpatients. Nonetheless, both clinical and microbiological evidence on the success of treatments, to date, are still scarce and limited to few studies and case reports mainly focusing on *Staphylococcus aureus*. Further studies are required in order to assess the safety and effectiveness of oritavancin in vascular, prosthetic, or device-related infections, as well as in other Gram-positive bacteria, especially enterococci.

## Figures and Tables

**Table 1:** Case series and case reports regarding oritavancin use outside of ABSSSI.

**Infective Endocarditis**						
**First Author** **et al. (Year) [Ref]**	**Type of Study**	**N. of Patients Treated, Type of Infection**	**Dosing and Interval**	**Pathogen (s)**	**Outcome**	**Adverse Effects**
Johnson et al. (2015) [12]	Case Report	1 (1), PVE	1200 q48h × 3; 1200 twice weekly for 6 wks; After recurrence 2 wks twice weekly in combination with gentamicin (4 days) and then linezolid and tigecicline; 10 wks after surgery 1200 twice weekly (in the first ten days in addition with linezolid and tigecycline)	VRE	Favourable after surgical valve replacement	Increased aPTT; nausea and anorexia (during combination therapy with linezolid and tigecycline)
Stewart et al. (2017) [13]	Case Series	1 (10), NVE	1200 single dose (after 3 days of vancomycin and 4 days of ceftriaxone)	Group B Streptococcus	Failure due to the need for surgical intervention and hospital readmission 3 months later	None
Salcedo et al. (2018) [15]	Case Series	5 (5), NVE	1200 single dose in 3 patiens, 1200 weekly × 4 in 2 patients	2 MSSA, 2 MRSA and 1 GBS/GFS	3 favourable. 2 Not reported	One patients reported eosinophilia/anaphylaxis
Brownell et al. (2020) [14]	Retrospective, observational	4 (75), Endocarditis not specified	NA	NA	Favourable in 100% of cases	NA
Morrisette et al. (2019) [16]	Retrospective, multicenter	1 (56), Endocarditis	1200 single dose, then lost to follow-up	*E. faecalis*	Lost to follow-up	NA
Ahiskali et al. (2020) [17]	Retrospective, observational	2 (24), Endocarditis not specified	1200 single dose in 1 patients, 1200 weekly × 2 in 1 patient	1 MSSA and 1 MRSA	Clinical cure (MSSA). Failure (complicated by spondylodiscitis and epidural abscess)	NA
**Device-Related Infections**						
**First Author** **et al. (Year) [Ref]**	**Type of Study**	**N. of Patients Treated**	**Dosing and Interval**	**Pathogen(s)**	**Outcome**	**Adverse Effects**
Stewart et al. (2017) [13]	Case Series	1 (10), CLABSI	1200 single dose (after 4 days of vancomycin and 1 days of cefazoline)	MSSA	Favourable	Nausea
Shulz et al. (2018) [18]	retrospective, observational	1 (17), endovascular graft infection	1200 mg × 1; 800 mg/wk × 11 doses; 1200 mg × 1 following 11-day intervals; and 800 mg × 5/wk	*S. lugdunensis*	Palliative intent	NA
Morrisette et al. (2019) [16]	Retrospective, multicenter	2 (56), CLABSI	NA	NA	NA	NA
Co et al. (2018) [19]	Retrospective, observational	7 (67), cardiac device infection	NA	NA	NA	NA
Brownell et al. (2020) [14]	Retrospective, observational	2 (75), Line infection	NA	NA	Favourable in 100% of cases	NA
Redell et al. (2019) [20]	Retrospective, observational	2 (440), 1 exit site infection, 1 Spinal Hardware	1200 single dose	NA	NA	NA
**Blood-Stream Infections**						
**First Author** **et al. [Ref]**	**Type of Study**	**N. of Patients Treated**	**Dosing and Interval**	**Pathogen(s)**	**Outcome**	**Adverse Effects**
Stewart et al. (2017) [13]	Case Series	6 (10), isolated BSI	1200 single dose	4 MSSA, 1 enterococcus (Ampicillin-susceptible) and 1 CoNS	4 Favourable, 1 Failure and 1 Not evaluable	None
Ahiskali et al. (2020) [17]	Retrospective, observational	3 (24), isolated BSI	1200 single dose in 5 patients, 1200 weekly × 2 in 2 patient, 1200 weekly × 4 in 1 patient	5 MRSA and 4 MSSA	3 complete cure, 4 incomplete cure, 2 lost at FUP	NA
Redell et al. (2019) [20]	Retrospective, observational	7 (440), isolated BSI	1200 single dose	NA	NA	NA
**Prosthetic Joint Infections**						
**First Author** **et al. [Ref]**	**Type of Study**	**N. of Patients Treated**	**Dosing and Interval**	**Pathogen(s)**	**Outcome**	**Adverse Effects**
Van Hise et al. (2020) [21]	multicenter, retrospective, descriptive	134 ostheomyelitis, of which 24 prosthetic	1200 mg, then 800 mg weekly (4 to 5 doses)	71.9% MRSA	88.1% clinical success at the end of therapy	3 hypoglycemia, 1 tachycardia, 1 tachycardia with chest pain
Redell et al. (2019) [20]	retrospective, observational	438, of which 18 osteomyelitis and 3 prosthetic	1200 mg every 6–14 days (1–10 doses)	74% *S. aureus* of which 59.3% MRSA	93.8% cure or 30-days improvement	6.6% of patients reported an adverse event
Shulz et al. (2018) [18]	retrospective, observational	17 including osteomyelitis	1200 mg (2–18 doses)	NA	100% clinical success or improvement	24% of patients reported an adverse event
Delaportas et al. (2017) [22]	case report	1 ostheomyelitis	1200 mg weekly (6 doses)	MSSA	clinical cure	NA
Chastain et al. (2018) [23]	case series	9 chronic ostheomyelitis	1200 mg—variable time between doses (2–6 doses)	5 MRSA	100% clinical cure at 6-months follow up	None
Nguyen et al. (2020) [24]	case report	1 prosthetic joint infection	daptomycin plus ampicillin 10 days, then 1200 mg weekly (6 doses)	vancomycin sensitive *E. faecalis*	clinical cure	NA
Dahesh et al. (2019) [25]	case report	1 prosthetic vertebral ostheomyelitis	1200 mg weekly (2 doses), then 800 mg weekly (8 doses) plus ampicillin	vancomycin-resistant *E. faecium*	clinical cure	NA

Abbreviations: Ref: references; BSI: bloodstream infections; CLABSI: central-line-associated BSI; NVE: native valve endocarditis; PVE: prosthetic valve endocarditis; MSSA: methicillin-susceptible *Staphylococcus aureus*; MRSA: methicillin-resistant Staphylococcus aureus; GBS: Group B Streptococcus; GFS: Group F Streptococcus; NA: not available; VRE: vancomycin-resistant enterococcus; PTT: prothrombin time.

## Data Availability

Not applicable.
